# Exploration of Microalgal Species for Nutrient Removal from Anaerobically Digested Swine Wastewater and Potential Lipids Production

**DOI:** 10.3390/microorganisms9122469

**Published:** 2021-11-30

**Authors:** Zhihui Chen, Yunhua Xiao, Tan Liu, Mingmin Yuan, Gang Liu, Jun Fang, Bo Yang

**Affiliations:** Hunan Provincial Engineering Research Center of Applied Microbial Resources Development for Livestock and Poultry, College of Bioscience and Biotechnology, Hunan Agricultural University, Changsha 410125, China; chenzhihui@stu.hunau.edu.cn (Z.C.); huazipiaoling.123@163.com (Y.X.); liutan@stu.hunau.edu.cn (T.L.); yuanmingmin@stu.hunau.edu.cn (M.Y.); gangle.liu@gmail.com (G.L.)

**Keywords:** wastewater treatment, microalgae, nutrient removal, lipid, biodiesel, *Chlorella*

## Abstract

Bio-treatment of anaerobically treated swine wastewater (ADSW) mediated by microalgae has been deemed as a promising strategy. In the present study, six microalgal strains were used to conduct batch experiments in 0~100% ADSW in order to evaluate their potentials for nutrient removal and biodiesel production. Two strains, *Chlorella vulgaris* FACHB-8 and *Chlorella* sp. FACHB-31, were selected based on their better growth performances, higher tolerance to wastewater (up to 100%), and better nutrient removal abilities. The capacity of each strain to remove TN, TP, NH_4_^+^-N, as well as lipid production and biomass composition in 100% ADSW were further examined. After 15 days of culture, 87.68~89.85%, 92.61~93.68%, and 97.02~97.86% of the nitrogen, phosphorus, and ammonia nitrogen were removed by *Chlorella* sp. FACHB-31 and *C. vulgaris* FACHB-8. Their lipid content and lipid productivities were 29.63~33.33% and 18.91~23.10 mg L^−1^ d^−1^, respectively. Proteins were both the major biomass fraction followed by lipids and then carbohydrates. Their fatty acid profiles both mainly consisted of C-16:0, C-18:1, C-18:0, and C-18:2. Taken together, our results suggest that *C. vulgaris* FACHB-8 and *Chlorella* sp. FACHB-31 are potential candidates for biodiesel production by using ADSW as a good feedstock.

## 1. Introduction

Environmental pollution has aroused extensive attention in the international community for many decades. Population growth and vast industrialization have led to several environmental problems, such as water pollution [1]. Swine slurry discharged into the aquatic environment has been regarded as the main source of water pollution, notably in China [2]. Swine wastewater contains high concentrations of nutrients such as nitrogen and phosphorus, as well as various trace elements such as K, Mg, Ca, etc. [3]. Hence, the discharge of it causes great damages to the ecological environment, particularly water eutrophication and harmful algal blooms [4]. This has prompted efforts to ameliorate the water quality by developing eco-friendly and sustainable strategies for wastewater treatment.

In recent years, the cultivation of microalgae using wastewater as a source of nutrients has been proposed as a feasible strategy for wastewater treatment and the production of renewable biosources [5]. The microalgae-based combined approach is considered as tertiary wastewater treatment [6]. Microalgae are sunlight-driven organisms that are capable of converting atmospheric carbon dioxide to various valuable bioactive compounds including lipids, proteins, carotenoids, vitamins, and minerals [7,8]. It is well known that microalgae can be grown in photoautotrophic, mixotrophic, and heterotrophic culture conditions. They are able to grow fast without competing for arable land and are easy to be cultured in controlled conditions to attain high cell density [7,9]. Besides, they have greater photosynthetic efficiency that is 10~50 times higher than terrestrial plants [10]. Reports have shown that many microalgae are exceedingly rich in oil and their lipid contents ranged from 47.9~72% [11]. The lipid productivity of microalgae is 15~300 times that of conventional crops [12]. In this regard, microalgae have emerged as a potential source for biodiesel production. In recent years, they have been considered as a third-generation biofuel feedstock that may replace traditional fossil fuels [13]. The use of microalgal biomass for biodiesel production is eco-friendly and sustainable. Combining microalgae cultivation with wastewater treatment is a cost-effective approach for biodiesel production, offsetting the cost of pollution control [14,15].

Many studies have shown the great potential of microalgae in removing nutrients from various types of wastewater and in reaching higher productivities [5,16,17,18]. Microalgae are able to use wastewater effluent as a source of carbon, nitrogen, and phosphorus to sustain their growth effectively. The selection of efficient algal species that have great cell growth and good tolerance to wastewater is a vital criterion in maximizing the benefit from microalgae [19]. Several algal species, including *Chlorella* and *Scenedesmus*, have been generally considered as promising candidates for wastewater treatment [17,20,21,22]. *S. obliquus* and *C. sorokiniana* have been shown to have good growth performances and nutrient removal abilities in domestic wastewater, with a daily nitrogen removal of 4.4 and 6.6 mg N L^−1^ d^−1^, respectively [23]. *S. obliquus* was found to have an NH_4_^+^-N removal efficiency of 83% after 21 days of culture in diluted livestock wastewater [24]. More recent studies have shown that *Scenedesmus* sp. had a high removal of TN, TP, NH_4_^+^-N (>95%) in simulated wastewater, with a biomass production of 7.2 × 10^6^ cells mL^−1^ and lipid productivity of 5.67 mg L^−1^ d^−1^ during 12-day cultivation [11]. In addition, a previous study has shown that *C. vulgaris* can be cultured in undiluted anaerobically treated swine wastewater in an open raceway pond, with a TN removal of 89.5% and TP removal of 85.3%, respectively [9]. Nonetheless, only a handful of microalgal strains can survive and grow well in real undiluted swine wastewater due to the high concentration of ammonia nitrogen and turbidity. Most of them were performed in diluted wastewater or simulated wastewater so as to reduce the toxicity of pollutants [25]. Thus, this presents a key bottleneck for culturing microalgae by using wastewater as a nutrient source. Accordingly, screening several microalgal species that can grow in swine wastewater with high nutrient levels is essential and imperative.

The aim of this work was intended to screen efficient algal strains for use in the treatment of anaerobically treated swine wastewater (ADSW) and as a potential source for biodiesel production. To our knowledge, biotreatment of ADSW by microalgae is rarely reported. As the anaerobic digestion process could consume organic nutrients and generate a high level of ammonia nitrogen, only a few microalgae can survive in ADSW. Hence, in our study, six different strains were first evaluated to grow in various dilutions of ADSW in terms of growth performance and nutrient removal ability. Afterward, two promising selected strains were studied in more detail by examining their removal efficiencies of total nitrogen (TN), total phosphorus (TP), and ammonia nitrogen (NH_4_^+^-N), and their capacities to produce lipids. The biochemical composition, including carbohydrate and protein content, and fatty acid profiles were also investigated. Our work demonstrates the great potential of using algal strain as a candidate for biodiesel production using ADSW as a feedstock.

## 2. Materials and Methods

### 2.1. Wastewater Collection, Pretreatment, and Analysis

Samples of anaerobically treated swine wastewater (ADSW) were collected from a local pig farm (Xinguang’ an Agriculture and Animal Husbandry Co., Ltd.) in Changsha, Hunan province, China. The original ADSW sample was centrifuged at 8000× *g* for 10 min and immediately filtered by 0.45 µm nylon membrane to remove insoluble solids. The wastewater was then autoclaved at 121 °C for 20 min. The pretreated liquid was stored at 4 °C until further use. For the physicochemical analysis of the wastewater, the pH value was measured using the glass electrode method. The total nitrogen (TN) level and total phosphorus (TP) level was determined by using UV spectrophotometry with alkaline potassium persulfate digestion and ammonium molybdate spectrophotometry, respectively. The NH_4_^+^-N level was determined by using NaCl reagent spectrophotometry. Chemical oxygen demand (COD) was measured using the dichromate method. Total organic carbon (TOC), inorganic carbon (IC), and heavy metal ions (As, Cd, Cr, Cu, Fe, Mn, Ni, Pb, Zn, Sb), were determined according to the Chinese National Standards. The compositions of original and autoclaved pretreated ADSW were shown in Table 1.

### 2.2. Strains, Culture Conditions and Microalgae Selection

Six microalgal strains, i.e., *Chromochloris zofingiensis* GF1, *Chlorella protothecoide* GF2, *Chlorella pyrenoidosa* GF3, *Chlorella vulgaris* FACHB-8, *Chlorella* sp. FACHB-31, *Scenedesmus obliquus* FACHB-417, were investigated in this experiment. The first three algae were kindly provided by Prof. Feng Chen, Shenzhen University. The other three algae were obtained from the Freshwater Algae Culture Collection at the Institute of Hydrobiology (FACHB). Algal strains were cultured and maintained in BG11 medium [26] consisting of (per liter) NaNO_3_ 1.5 g, K_2_HPO_4_ 0.04 g, MgSO_4_·7H_2_O 0.075 g, CaCl_2_·2H_2_O 0.036 g, Na_2_CO_3_ 0.02 g, ferric ammonium citrate 6.00 mg, citric acid·H_2_O 6.00 mg, Na_2_EDTA·2H_2_O 1.00 mg, H_3_BO_3_ 2.86 mg, MnCl_2_·4H_2_O 1.81 mg, ZnSO_4_·7H_2_O 0.22 mg, Na_2_MoO_4_·2H_2_O 0.39 mg, CuSO_4_·5H_2_O 0.079 mg, Co(NO_3_)_2_·6H_2_O 0.0494 mg. The pH of the medium was adjusted to 7.2 prior to autoclaving.

In all cases, 130 mL of the liquid medium in 250-mL Erlenmeyer flasks were inoculated at 10% (*v*/*v*). The cultures were incubated at 80 μmol photon m^−2^ s^−1^ under a photoperiod of 12:12-h light/dark cycle at 25 °C with orbital shaking at 150 rpm. For the experiment with respect to algal strain selection, the autoclaved pretreated ADSW was diluted with distilled water (*v*/*v*) to five various concentrations at a level of 20%, 40%, 60%, 80%, and 100% ADSW. The pH values of five dilutions of ADSW were all adjusted to 7.2 prior to autoclaving. All the initial cell densities were adjusted to approximately 2 × 10^7^ cells per mL. The algal cells were cultured for 14 days. For the experiment concerning nutrient removal of two selected strains, algal strains were cultured in undiluted ADSW (100% ADSW) with an initial cell density of 2 × 10^7^ cells per mL. All the culture conditions were the same as described above except the algal cells were cultured for 15 days.

### 2.3. Measurement of Cell Growth

The dry cell weight (g L^−1^) was determined according to the method as described by [8]. Cells were sedimented by centrifugation and washed twice with distilled water. Then the biomass was determined after drying in the oven at 80 °C overnight.

Biomass productivity (g L^−1^ d^−1^) during the culture period was calculated from the following Equation (1):

*P* = (*X*_1_ − *X*_0_)/(*t*_1_
*− t*_0_) (1)

Specific growth rate *µ* (d^−1^) was calculated from the following Equation (2):

*µ* = (ln*X*_1_ − ln*X*_0_)/(*t*_1_
*− t*_0_) (2)

where *X*_1_ and *X*_0_ (g L^−1^) were the biomass concentration on days *t*_1_ and *t*_0_, respectively.

### 2.4. Nutrient Removal Analysis

A volume of 5 mL culture suspension was collected from each flask for nutrient removal analysis. The samples were centrifuged at 9000× *g* for 10 min, and the supernatants were appropriately diluted and analyzed for TN, TP, COD, and NH_4_^+^-N as described in Section 2.1.

### 2.5. Determination of Biochemical Composition of Algal Cells

For pigment analysis, chlorophyll *a* in harvested algal cells was extracted with 1 mL of methanol for 5 h 4 °C and then centrifuged, the absorbance of the supernatant was measured at 750 nm, 665 nm, 652 nm with an ultraviolet-near-infrared spectrophotometer UV-3600 Plus (Shimadzu, Kyoto, Japan). The pigment concentrations were calculated according to the following equations [27].

[Chlorophyll *a*] mg/L = 16.5169 × A_665_ − 8.0962 × A_652_

The absorbance at 652 nm and 665 nm is corrected by subtracting the absorbance at 750 nm.

For carbohydrate content, 10 mg of lyophilized algal biomass was extracted with distilled water in a boiling water bath for 30 min (3 times). The extract was filtered into a 25-mL volumetric flask and diluted to volume with distilled water. The total carbohydrate was measured using the phenol-sulfuric method [28]. For protein content, briefly, 200 μL of 1 M NaOH was added to 10 mg of lyophilized algal biomass and hydrolyzed for 10 min at 80 °C. Then 1.8 mL of distilled water was added to the hydrolysate and centrifuged at 12,000× *g* for 30 min. The supernatant was collected into a new centrifuge tube. Each biomass sample was extracted twice and the supernatants were collected together. Finally, the protein content was determined using the Protein analysis kit (Bradford P0006, Beyotime, Shanghai, China).

### 2.6. Lipid and Fatty Acid Analysis

Total lipids were extracted from lyophilized algal biomass by using a solvent-based method deriving from Kumar et al. [15] and determined gravimetrically. Briefly, 20 mg of lyophilized algal powder was weighed into a preweighted 10 mL centrifuge tube, and 3 mL of methanol-chloroform (2:1, *v*/*v*) mixture was added. The tube was fully mixed for 2 min and then centrifuged at 10,000× *g* for 5 min. The chloroform layer was collected, and this extract process was repeated three times. The collected chloroform layer was evaporated under nitrogen gas and dried in a dry oven at 80 °C until constant weight and followed by gravimetrical measurement of the residues.

Fatty acids were analyzed by using a modification to the method as described by Wang et al. [9]. Twenty milligrams of lyophilized algal biomass were suspended with solvent mixtures (1 mL of toluene, 2 mL of 1% methanol sulfate (*v*/*v*), and 0.8 mg of heptadecanoic acid at 0.8 mL of hexane internal standard) overnight to form fatty acid methyl esters (hunger) at 50 °C. Then hexane was extracted three times in a reciprocating vibrating screen. Fatty acid methyl esters (FAMEs) in the extracted liquid were quantified by a QP 2010 SE gas chromatography-mass spectrometer (Shimadzu, Japan) using a Stabilwax-DA capillary column (30 m × 0.25 mm × 0.25 μm) (Shimadzu, Japan). Injection temperature was 250 °C, and the initial column temperature was set at 150 °C and then the temperature was increased to 200 °C at 10 °C/min and then to 220 °C at 6 °C/min, followed by a hold at 220 °C at 10 min. FAMEs were identified by comparing their fragmentation patterns with those in the NIST mass spectral library.

### 2.7. Statistical Analysis

In this study, SPSS and GraphPad software were used for data processing. The measured value was expressed as the mean ± standard deviation (SD). Analysis of variance (ANOVA) and *t*-tests was implemented via SPSS software (version 19.0) wherever applicable. A confidence level of 95% was selected in order to strictly determine the significance. There was a statistically significant difference when *p* < 0.05.

## 3. Results and Discussion

### 3.1. Selection of Microalgae Based on Growth Performance

Microalgae can grow rapidly under favorable culture conditions with an adequate supply of nutrients. Swine wastewater consists of vital macronutrients such as nitrogen and phosphorous for algal growth and thus can be used as a promising feedstock for cultivating microalgae [5]. In most cases, wastewater samples were autoclaved in order to reduce the possible impact of the microbial community in wastewater before being prepared as a culture medium. Numerous studies have utilized various sources of autoclaved wastewater to cultivate microalgal strains on a laboratory scale [9]. In this regard, the autoclaved ADSW was adopted as a culture medium in this work. The physicochemical properties of non-autoclaved and autoclaved ADSW are listed in Table 1. It was obviously shown that the sterilization process attenuated the TN, TP, NH_4_^+^-N, and COD levels of wastewater compared with the non-autoclaved ones. The concentrations of TN, TP, NH_4_^+^-N, and COD were decreased to 258.5, 65.75, 183.5, and 900.01 mg L^−1^, respectively (Table 1). This is in accordance with results observed in other autoclaved swine wastewaters [9,29]. Besides, the concentrations of toxic metals in ADSW were very low (Table 1), suggesting that the wastewater used was not likely to be toxic to algal cells.

In addition to being subjected to the autoclaved process, wastewater is usually diluted before being fed to microalgal cells. This is owed to a very high level of nutrients (e.g., >100 mg/L NH_4_^+^-N concentration) in wastewater that may inhibit algal growth [30,31]. This would lead to a low nutrient removal efficiency and biomass productivity. Hence in our work, all algal strains were firstly inoculated in varying dilutions of ADSW (20%, 40%, 60%, 80%, 100% ADSW) in order to test whether they could grow in high levels of ADSW and evaluate the potential of practical use in wastewater treatment.

Green algae are usually highly tolerant to different kinds of contaminants, including organic compounds, ammonia, and heavy metals [11]. Therefore, in most cases they can be cultured in wastewater, utilizing different contaminants as nutrient sources and thereby purifying the wastewater [14,32]. In addition, several green algal species, such as *Chlorella* and *Scenedesmus*, are able to be cultured to accumulate significant amounts of value-added lipids, showing great potential for biodiesel production [33]. As a result, in our study, we selected six commonly used algal species to test the potential of practical use in wastewater treatment and simultaneous lipid production. All six microalgal strains are generally regarded as green algae and belong to *Chlorophyta*. Among these selected strains, *S. obliquus* FACHB-417, *C. vulgaris* FACHB-8, and *Chlorella* sp. FACHB-31 has been demonstrated their aptitude to be employed as biomass producers from wastewater sources [29,34]. This suggests a possible potential use in our study for ADSW treatment. The other three selected strains, *C. zofingiensis* GF1, *C. protothecoide* GF2, *C. pyrenoidosa* GF3, to our best knowledge, have not yet been reported in wastewater treatment and therefore examined in this study.

**Figure 1:**
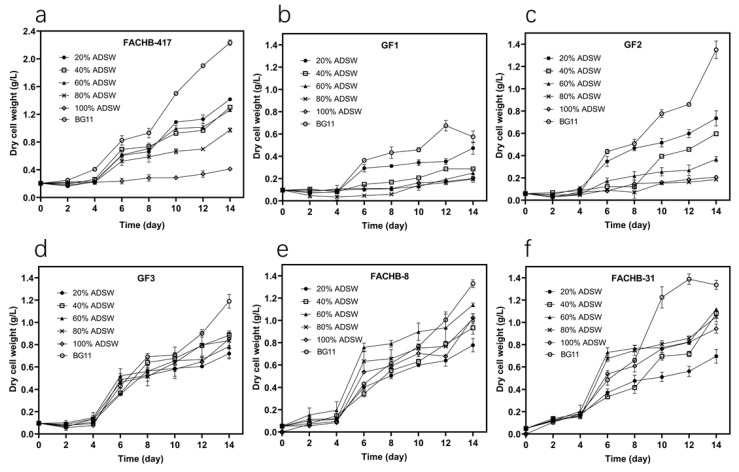
Growth curves for six microalgal strains in five dilutions of ADSW and BG11 medium within 14 days. (**a**) *S. obliquus* FACHB-417, (**b**) *C. zofingiensis* GF1, (**c**) *C. protothecoides* GF2, (**d**) *C. pyrenoidosa* GF3, (**e**) *C. vulgaris* FACHB-8, and (**f**) *Chlorella* sp. FACHB-31. Error bars represent SD (*n* = 3).

The growth curves of six strains at five dilutions of ADSW and BG11 medium within 14 days are represented in Figure 1. The biomass production, biomass productivity, maximum biomass productivity, specific growth rate, and maximum specific growth rate for all strains in all samples of ADSW and BG11 medium are compared in Table 2. BG11 medium enriches various kinds of algal strains and was used as a control for culturing all tested strains in this study. As shown in Figure 1, all strains exhibited a lag phase during the first four days in all dilutions of ADSW and then began to enter the exponential phase. After 14 days of incubation in ADSW, *S. obliquus* FACHB-417 exhibited the highest biomass production in 20% ADSW (1.417 g L^−1^), followed by *C. vulgaris* FACHB-8 in 60% ADSW (1.140 g L^−1^), *Chlorella* sp. FACHB-31 in 60% ADSW (1.118 g L^−1^), *C. pyrenoidosa* GF3 in 40% ADSW (0.885 g L^−1^), *C. protothecoides* in 20% ADSW (0.735 g L^−1^) and *C. zofingiensis* GF1 in 20% ADSW (0.473 g L^−1^) (Table 2). As expected, all strains grow faster in the BG11 medium than in all five dilutions of ADSW based on a comparison of specific growth rates (Table 2). This lies in the fact that the nutrients in different dilutions of ADSW are lower than that in the BG11 medium. The dark color of undiluted ADSW may also have a certain impact on cell growth. Besides, this also indicated that the adaptability of our selected strains may not be good enough to easily adapt to the special ecological environment. Reports have revealed that indigenous microalgae isolated from water bodies or special natural environments could easily adjust their physiochemical traits to adapt better under specific environmental conditions [20,29]. As all of our strains were not naturally screened from swine slurry, this might as well probably lead to a lower growth rate in ADSW than in the BG11 medium.

As presented in Figure 1, almost all strains could grow well in lower dilutions (20% and 40% ADSW) except GF1. Notably, GF1 showed a much poor growth performance in all dilutions of ADSW as compared to the other five strains. The biomass production and productivity of GF1 in 20% ADSW were the highest, correspondingly to 0.473 g L^−1^ and 26.905 mg L^−1^ d^−1^, much lower than the highest data observed in the other strains (Table 2). Besides, GF1 showed a very low biomass production in undiluted raw ADSW (100% ADSW). These suggest that GF1 has poor environmental adaptability to ADSW and has little potential for practical use in wastewater treatment.

For each strain of FACHB-417, GF1, and GF2, compared with the serial dilutions, the biomass productivity and specific growth rate almost decreased with the increase of the ADSW concentration after 14 days of culture (Table 2). In other words, almost all of these three strains showed growth inhibition in higher concentrations of ADSW (>60%). For example, FACHB-417 grew fast in lower concentrations of ADSW (≤60%) and yielded the highest biomass of 1.417 g L^−1^ in 20% ADSW. However, it did not have good tolerance and grew slowly in higher concentrations of ADSW (>60%), especially with the lowest biomass of 0.412 g L^−1^ in 100% ADSW. Similar results were also observed in the cultivation of *Coelastrella* sp. and *C. vulgaris* using swine wastewater as a culture medium [20,35]. The possible reason for this lies in that the relatively high levels of COD and NH_4_^+^-N in high concentrations of ADSW may inhibit the growth of algal cells. In our study, our purpose was to select a promising strain that has great potential for the treatment of undiluted raw ADSW and for biodiesel production. Consequently, the strain selected in our work must have good tolerance to high concentrations of ADSW and can thereby grow well and remove nutrients from raw ADSW. As readily seen in Table 2, FACHB-417, GF1, and GF2 grew poorly in undiluted raw ADSW (100% ADSW) and could only produce very little biomass. This illustrates that these three strains cannot adapt well to high concentrations of ADSW and may not be suitable for wastewater treatment.

It could be observed from Table 2 that, GF3, FACHB-8, and FACHB-31 could all grow well when cultured in all tested dilutions of ADSW (Figure 1 and Table 2), showing good adaptability to ADSW. Almost the higher the ADSW concentration was, the better the strain performed. After 14 days of incubation in 80% and 100% ADSW, GF3, FACHB-8, and FACHB-31 showed biomass productivity of 0.053~0.054, 0.069~0.069, and 0.064~0.071 g L^−1^ d^−1^, respectively, and biomass production of 0.837~0.852, 1.015~1.023, 0.943~1.050 g L^−1^, respectively. Therefore, the growth performances of FACHB-8 and FACHB-31 were better than that of GF3 in higher concentrations of ADSW. The highest specific growth rate was obtained in 100% ADSW for FACHB-8 (0.249 d^−1^) and in 80% ADSW for FACHB-31 (0.192 d^−1^). Besides, both FACHB-8 and FACHB-31 had a much higher maximum biomass productivity and maximum specific growth rate in higher concentrations of ADSW (≥60%) than in lower concentrations of ADSW (<60%), demonstrating a good tolerance to ADSW. These results were in accordance with previous reports that both FACHB-8 and FACHB-31 have good potentials for wastewater treatment [29,36].

**Figure 2:**
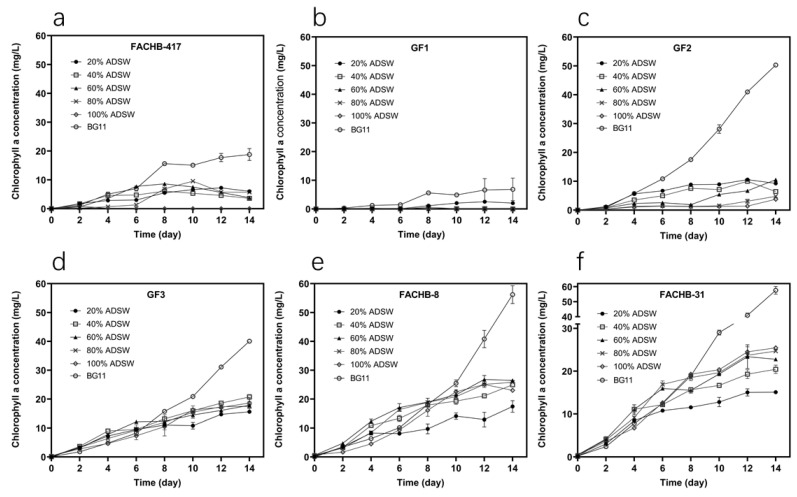
Time course of chlorophyll *a* concentration for six microalgal strains in five dilutions of ADSW and BG11 medium within 14 days. (**a**) *S. obliquus* FACHB-417, (**b**) *C. zofingiensis* GF1, (**c**) *C. protothecoides* GF2, (**d**) *C. pyrenoidosa* GF3, (**e**) *C. vulgaris* FACHB-8, and (**f**) *Chlorella* sp. FACHB-31. Error bars represent SD (*n* = 3).

Chlorophyll *a* is the principal pigment in aquatic microalgae and is generally regarded as an estimator of phytoplankton biomass in numerous studies [36]. In our study, chlorophyll *a* concentration was also measured to add the comparability of various algal species. As expected, the chlorophyll *a* concentration of FACHB-417, GF1, and GF2 almost decreased with the increase of the ADSW concentration after 14 days of culture (Figure 2). Compared to the other strains, both FACHB-8 and FACHB-31 had a much higher chlorophyll *a* concentration in high concentrations of ADSW (Figure 2). These results were in accordance with the above-mentioned growth data, indicative of the better growth performances of FACHB-8 and FACHB-31 in high concentrations of ADSW among six strains. Taken together, considering all factors listed in Table 2, FACHB-8 and FACHB-31 were the best candidates for growing in undiluted raw ADSW.

### 3.2. Selection of Microalgae Based on Nutrient Removal Ability

After 14 days of incubation, the removal efficiencies of TN, TP, and NH_4_^+^-N by six strains cultured in five dilutions of ADSW were studied and the results were listed in Table 3. For FACHB-417, GF1, and GF2, the removal of TN was higher in low dilutions (20~40% ADSW) than in high dilutions (60~100% ADSW). The highest removal of TN for FACHB-417, GF1 and GF2 was found in 40% ADSW (82.89%), 20% ADSW (74.87%) and 40% ADSW (83.51%), respectively. While for GF3, FACHB-8, and FACHB-31, the TN removal all enhanced with an increase in ADSW concentration from 0% to 100%. In undiluted ADSW (100% ADSW), FACHB-8 and FACHB-31 showed the highest TN removal of 84.21% and 84.24%, respectively, followed by 79.64% for GF3. These results indicate that both FACHB-8 and FACHB-31 have better removal ability of TN (~84%) compared to the other strains. Microalgae usually have good adaptability and therefore can be cultured in wastewater for biomass production by using the nutrients present in wastewater [37]. They require large amounts of nitrogen and phosphorus for cell growth. Nitrogen plays a significant role in facilitating cell growth. Eukaryotic microalgae are able to assimilate fixed nitrogen, such as NH_4_^+^-N, NO_3_^−^-N and etc., [38]. They can utilize nitrogen to build proteins, nucleic acids, among other molecules.

Concerning phosphorus, it also plays an important role in microalgae growth and metabolism as a component of nucleic acids, adenosine triphosphate, phospholipids and etc., [38]. Table 3 showed the removal of TP in various dilutions of ADSW. When the cultivations came to an end, nearly 90% of TP was removed for all strains in all trials except GF1. The higher TP removal obtained in our study is similar to previous studies [20]. While GF1 showed the lowest TP removal of less than 70% in all dilutions of ADSW, which was proportional to its growth performance as mentioned above (Table 2). In addition, the TP removal of all strains was almost greater in low dilutions than in high dilutions except GF1 (Table 3). The highest TP removal was observed for GF2 in 40% ADSW (97.57%) and was followed by FACHB-417 in 20% ADSW (97.19%), FACHB-31 in 40% ADSW (96.35%), FACHB-8 in 40% ADSW (95.39%), GF3 in 40% ADSW (94.94%), and GF1 in 100% ADSW (69.94%). Comparatively, phosphorus could be reduced more efficiently than nitrogen for almost all strains. Noteworthy, the removal of TP was not analogous to the above-mentioned growth performance. A similar finding was also reported by Aravantinou et al. [12].

NH_4_^+^-N was the main form of available nitrogen found in wastewater and was the preferred nitrogen source for algal growth [39]. The removal of NH_4_^+^-N during the period of 14 days is also shown in Table 3. All six strains showed a higher NH_4_^+^-N removal in low dilutions (20~40% ADSW) than in high dilutions (60~100% ADSW). In 100% ADSW, the NH_4_^+^-N removal of FACHB-417 was only 67.44%. Considering the poor growth performance (Table 2), as well as low removal of TN and TP in 100% ADSW as mentioned above (Table 3), it can be inferred that this strain cannot adapt well in raw ADSW and may not be suitable for wastewater treatment. It can be observed that even the initial NH_4_^+^-N concentration reaches 183.5 mg L^−1^ (Table 1), the NH_4_^+^-N removal of FACHB-8 and FACHB-31 in undiluted ADSW (100% ADSW) was relatively high (>90%) (Table 3). This indicated that both strains have good potentials for NH_4_^+^-N removal. The higher removal of NH_4_^+^-N in our work is similar to previous reports [11].

Overall, in our study, both FACHB-8 and FACHB-31 showed a satisfied removal ability of TN (~84%), TP (>90%), and NH_4_^+^-N (>90%) in undiluted raw ADSW. Considering their good performances in the same condition as mentioned above, FACHB-8 and FACHB-31 were therefore chosen among six strains for further study. Moreover, it can be speculated that the removal abilities of nitrogen and phosphorus of these two strains were analogous to their algal biomass, indicating that the abundant nutrients in wastewater sustain algal growth. This is in accordance with the results observed in *Chlorella* and *Scenedesmus* [40].

### 3.3. Nutrient Removal Ability of Two Selected Strains in Undiluted ADSW

**Figure 3:**
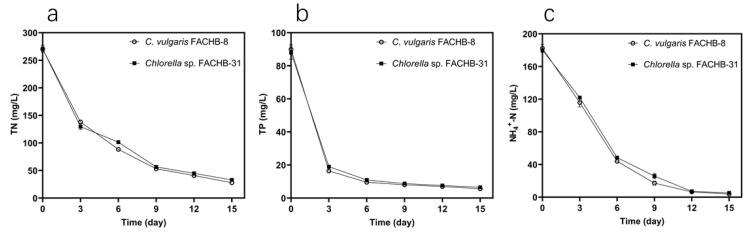
Changes in the removal of TN (**a**), TP (**b**), and NH_4_^+^-N (**c**) with time by FACHB-8 and FACHB-31 in undiluted ADSW within 15 days. Error bars represent SD (*n* = 3).

In order to examine the nutrient removal abilities of FACHB-8 and FACHB-31 in more detail, changes in the removal of TN, TP, and NH_4_^+^-N with time by these two strains in undiluted ADSW were studied. As shown in Figure 3, both strains showed a very similar trend of nutrient removal. The dynamic of TN, TP, and NH_4_^+^-N changes was that they were prone to be removed quickly in the first six days of culture, and then the rates of removal started to slow down. The TN removal of both strains was over 50% on the third day and reached over 80% on the ninth day (Figure 3a). At the end of cultivation, the TN removal of FACHB-8 and FACHB-31 was 89.85% and 87.68%, respectively. The TN concentration in ADSW by both strains after 15 days of culture was lower than 33 mg L^−1^.

While for phosphorus uptake, both strains showed a surprising TP removal of nearly 80% on the third day and approximately 90% on the sixth day (Figure 3b). This result is similar to the observations reported previously [9,40,41]. The possible reason for the dramatic removal of phosphorus in our study is not only the phosphorus assimilation by algal cells through metabolic pathways but also the external environmental conditions. Previous reports have shown that phosphorus precipitation may occur for pH values above 8.0 and high oxygen concentrations [38]. Hence, the increase of pH values in the early stage of culture (data not shown) might probably contribute to phosphorus precipitation and phosphate adsorption on microalgal cells. Thus, this further led to a dramatic TP removal in the first few days of our study. In the last nine days, the removal efficiency tended to be flat. Finally, FACHB-8 and FACHB-31 showed a TP removal of 93.68% and 92.61%, respectively, with the final TP concentrations of 5.68 and 6.50 mg L^−1^, respectively. There have been many studies reporting that the removal efficiencies of TN and TP by microalgae in wastewater correspond to 60~99% and 54~95%, respectively [38]. Hence, the removal efficiencies of TN and TP by both FACHB-8 and FACHB-31 obtained in our study were satisfied. As shown in Figure 3c, the NH_4_^+^-N removal of both strains was over 70% on the sixth day and even over 90% on the ninth day. After 15 days of culture, the NH_4_^+^-N concentration in ADSW by FACHB 8 and FACHB-31 was 3.91 and 5.36 mg L^−1^, with an NH_4_^+^-N removal of 97.86% and 97.02%, respectively.

Figure 3 shows that most phosphorous was consumed (>90%) before nitrogen and NH_4_^+^-N were exhausted after six days of cultivation. This suggests that phosphorus was the limiting factor in our study. This finding is similar to the results obtained in previous studies [42]. Phosphorus limitation may probably make microalgae grow slowly and thus have a negative impact on the removal of nitrogen. This may explain the reason why the total nitrogen in ADSW could not be removed as efficiently as TP and NH_4_^+^-N in our study. From the data obtained from Figure 3, it can be calculated that the amount of ammonium removal (nearly 180 mg L^−1^) accounted for the majority of the TN removal (nearly 240 mg L^−1^) for both FACHB-8 and FACHB-31. This implies that ammonium was the main nitrogen source consumed by both strains, in line with those ever reported in the treatment of swine wastewater and synthetic sewage by *Chlorella* and *Scenedesmus* [9,20].

In general, both FACHB-8 and FACHB-31 exhibited a higher nutrient removal capacity in undiluted ADSW with a TN removal of 87.68~89.85%, TP removal of 92.61~93.68%, and NH_4_^+^-N removal of 97.02~97.86%, respectively. This indicates that both *Chlorella* strains are promising candidates for swine wastewater treatment. Comparatively, FACHB-8 had a greater nutrient removal ability than FACHB-31 did. In our study, these two strains were chosen for further study of their potential for simultaneous lipid production.

### 3.4. Lipid Production, Biomass Composition, and FAME Composition

Microalgae can grow in wastewater using different pollutants as nutrient sources and produce significant amounts of value-added bioactive compounds such as lipids, proteins, vitamins, and etc. This is an eco-friendly and cost-effective strategy, offsetting the cost of wastewater treatment [32,38]. Reports have shown that many *Chlorella* species are capable of accumulating high amounts of lipids in controlled conditions [33]. Therefore, the lipid production and lipid productivity of both *Chlorella* strains, FACHB-8 and FACHB-31, were investigated to examine whether they could remove nutrients with the simultaneous efficient production of lipids in undiluted ADSW. After 15 days of culture in raw ADSW, the total lipid content of FACHB-8 and FACHB-31 was 33.33% DCW, and 29.63% DCW, respectively. The corresponding lipid productivity of FACHB-8 and FACHB-31 was 18.91 and 23.10 mg L d^−1^, respectively. The lipid content obtained in our study was higher than that in previous reports, in which the lipid content of 14~26% was observed in *Chlorella* species using different wastewater sources [8,23,43]. The possible reason may result from that the phosphorus limitation led to high lipid content in microalgae [39], which was also observed in our study as mentioned above.

**Figure 4:**
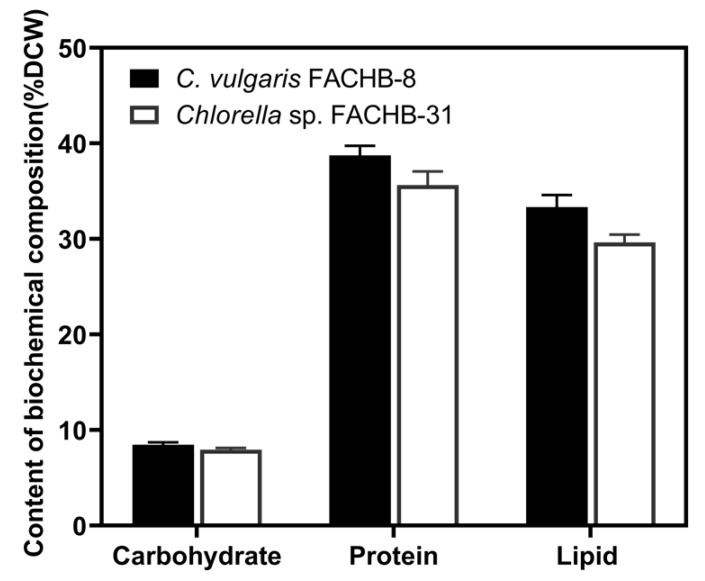
Comparison of content of carbohydrate, protein, and total lipid from FACHB-8 and FACHB-31 after 15 days of cultivation in undiluted ADSW. Error bars represent SD (*n* = 3).

The biomass composition of FACHB-8 and FACHB-31 after 15 days of cultivation in undiluted ADSW is presented in Figure 4. Except for lipids as mentioned above (29.63~33.33%), higher amounts of proteins were also accumulated in FACHB-8 (38.74% DCW) and FACHB-31 (35.64% DCW), similar to those achieved in domestic sewage [44]. However, low carbohydrate contents were obtained in these two strains (8.47% DCW for FACHB-8, and 7.94% DCW for FACHB-31). Comparatively, FACHB-8 exhibited a relatively better performance in the production of lipid, protein, and carbohydrate than FACHB-31 did. For both strains, proteins were the major biomass fraction followed by lipids and then carbohydrates.

The fatty acid profile determines the quality of microalgal biodiesel. The lipids extracted from FACHB-8 and FACHB-31 were esterified and the fatty acid composition of each strain was measured using gas chromatography. Their fatty acid profiles are detailed in Table 4. The main fatty acid components of the two strains were both fatty acids with C16~C18, accounting for approximately 85% of total fatty acids. This finding was comparable to previous results (80~90%) [39,40]. The dominant components in the two strains were palmitic acid (C16:0), oleic acid (C18:1), stearic acid (C18:0), and linoleic acid (C18:2), which was 27.46~27.95%, 20.25~20.57%, 17.29~17.59%, and 16.61~17.27% of total fatty acids, respectively. This is in accordance with the result observed in *Botryococcus braunii* using domestic sewage [44]. FACHB-8 and FACHB-31 had roughly equal quantities of saturated fatty acids (SFA) and unsaturated fatty acids (UFA), with 42.59~43.26% and 56.74~57.41%, respectively. Reports have shown that microalgae with approximately equal amounts of SFA and UFA are considered to have better performance in biodiesel production due to their oxidative stability and low-temperature characteristics [42]. Therefore, the microalgal lipids produced by FACHB-8 and FACHB-31 in our study are considered suitable for biodiesel production. It is well known that culture conditions (e.g., light intensity, temperature, air flow rate) and fermentation process may have a significant impact on biomass production, lipid production, and fatty acid composition. To promote lipid productivity and optimize the fatty acid profile, future research may lie in the exploration of the effects of culture conditions on cell growth, nutrient uptake, and lipid productivity of FACHB-8 and FACHB-31 using ADSW as a nutrient source.

## 4. Conclusions

Six microalgal strains were used to cultivate in 0~100% ADSW to evaluate their potentials for biomass production and nutrient removal. Both *C. vulgaris* FACHB-8 and *Chlorella* sp. FACHB-31 adapted quickly in all dilutions of ADSW and showed a much higher growth rate and better nutrient removal ability than the other strains did. After 15 days of cultivation in undiluted ADSW, both strains showed a TN removal of 87.68~89.85%, TP removal of 92.61~93.68%, and NH_4_^+^-N removal of 97.02~97.86%, respectively. The lipid content (29.63~33.33%), lipid productivity (18.91~23.10 mg L^−1^ d^−1^), and fatty acid methyl ester profiles of these two strains reinforce their potential use for biodiesel production. Overall, two *Chlorella* strains, FACHB-8 and FACHB-31 could be used as promising candidates for biodiesel production by using ADSW as a good feedstock.

## Figures and Tables

**Table 1:** Compositions of non-autoclaved and autoclaved ADSW.

Parameters	Non-Autoclaved ADSW	Autoclaved ADSW
TN (mg·L^−1^)	341.50 ± 9.71	258.50 ± 6.36
TP (mg·L^−1^)	80.75 ± 0.49	65.75 ± 0.64
NH_4_^+^-N (mg·L^−1^)	238.50 ± 4.95	183.50 ± 2.12
COD (mg·L^−1^)	1124.01 ± 23.46	900.01 ± 13.53
TOC (mg·L^−1^)	393.80 ± 10.87	194.80 ± 6.41
IC (mg·L^−1^)	324.12 ± 12.96	191.92 ± 7.05
As (mg·L^−1^)	0.00	0.00
Cd (mg·L^−1^)	0.00	0.00
Cr (mg·L^−1^)	0.86	0.65
Cu (mg·L^−1^)	0.21	0.03
Fe (mg·L^−1^)	3.66	2.19
Mn (mg·L^−1^)	0.45	0.18
Ni (mg·L^−1^)	0.16	0.08
Pb (mg·L^−1^)	3.90	3.90
Zn (mg·L^−1^)	0.12	0.05
Sb (mg·L^−1^)	0.005	0.004
pH	7.35 ± 0.21	8.36 ± 0.34

**Table 2:** Biomass production, biomass productivity, maximum biomass productivity, specific growth rate, maximum specific growth rate of six microalgal strains in five dilutions of ADSW and BG11 medium within 14 days.

Algal Species	Culture Medium	Biomass Production (g L^−1^)	Biomass Productivity (mg L^−1^ d^−1^)	Maximum Biomass Productivity (mg L^−1^ d^−1^)	Specific Growth Rate (d^−1^)	Maximum Specific Growth Rate (d^−1^)
*S*. *obliquus* FACHB-417	20% ADSW	1.417 ± 0.016 ^b^	86.548 ± 0.546 ^b^	213.333 ± 17.559 ^b^	0.182 ± 0.009 ^b^	0.250 ± 0.031 ^c^
	40% ADSW	1.303 ± 0.010 ^c^	78.453 ± 1.148 ^c^	216.667 ± 17.017 ^b^	0.161 ± 0.012 ^bc^	0.490 ± 0.053 ^a^
	60% ADSW	1.262 ± 0.025 ^c^	75.476 ± 2.431 ^c^	212.500 ± 18.284 ^b^	0.169 ± 0.021 ^bc^	0.485 ± 0.037 ^a^
	80% ADSW	0.973 ± 0.031 ^d^	54.881 ± 2.508 ^d^	166.250 ± 8.839 ^c^	0.147 ± 0.007 ^c^	0.425 ± 0.044 ^ab^
	100% ADSW	0.412 ± 0.010 ^e^	14.762 ± 1.443 ^e^	26.250 ± 5.303 ^d^	0.063 ± 0.011 ^d^	0.068 ± 0.010 ^d^
	BG11 medium	2.233 ± 0.039 ^a^	144.881 ± 3.037 ^a^	284.167 ± 33.572 ^a^	0.362 ± 0.009 ^a^	0.353 ± 0.037 ^bc^
*C*. *zofingiensis* GF1	20% ADSW	0.473 ± 0.055 ^b^	26.905 ± 4.306 ^b^	106.667 ± 14.216 ^b^	0.178 ± 0.012 ^ab^	0.650 ± 0.054 ^b^
	40% ADSW	0.287 ± 0.015 ^c^	13.571 ± 1.429 ^c^	41.667 ± 10.104 ^c^	0.125 ± 0.025 ^bc^	0.163 ± 0.042 ^d^
	60% ADSW	0.250 ± 0.035 ^cd^	10.953 ± 2.909 ^cd^	42.500 ± 3.536 ^c^	0.088 ± 0.030 ^c^	0.286 ± 0.018 ^c^
	80% ADSW	0.195 ± 0.028 ^d^	7.024 ± 1.967 ^d^	40.000 ± 11.456 ^c^	0.185 ± 0.075 ^ab^	0.348 ± 0.037 ^c^
	100% ADSW	0.202 ± 0.015 ^d^	7.500 ± 1.237 ^d^	16.250 ± 5.303 ^d^	0.074 ± 0.013 ^c^	0.156 ± 0.010 ^d^
	BG11 medium	0.575 ± 0.053 ^a^	33.929 ± 3.763 ^a^	140.833 ± 10.104 ^a^	0.195 ± 0.003 ^a^	0.748 ± 0.064 ^a^
*C*. *protothecoides* GF2	20% ADSW	0.735 ± 0.068 ^b^	48.214 ± 4.831 ^b^	123.333 ± 28.759 ^b^	0.199 ± 0.020 ^b^	0.6863 ± 0.294 ^a^
	40% ADSW	0.597 ± 0.018 ^c^	38.333 ± 1.254 ^c^	136.667 ± 3.819 ^b^	0.194 ± 0.015 ^b^	0.589 ± 0.025 ^b^
	60% ADSW	0.368 ± 0.024 ^d^	22.024 ± 1.688 ^d^	60.833 ± 11.273 ^c^	0.196 ± 0.011 ^b^	0.602 ± 0.049 ^b^
	80% ADSW	0.185 ± 0.013 ^e^	8.929 ± 0.945 ^e^	25.000 ± 5.000 ^d^	0.138 ± 0.018 ^c^	0.358 ±0.036 ^c^
	100% ADSW	0.207 ± 0.003 ^e^	10.476 ± 0.206 ^e^	25.833 ± 1.443 ^d^	0.105 ± 0.019 ^d^	0.310 ± 0.048 ^c^
	BG11 medium	1.350 ± 0.079 ^a^	92.143 ± 5.669 ^a^	188.333 ± 12.583 ^a^	0.311 ± 0.009 ^a^	0.225 ± 0.023 ^d^
*C. pyrenoidosa* GF3	20% ADSW	0.720 ± 0.048 ^c^	44.524 ± 3.079 ^c^	123.333 ± 10.408 ^d^	0.188 ± 0.013 ^b^	0.591 ± 0.073 ^c^
	40% ADSW	0.885 ± 0.040 ^b^	56.309 ± 3.221 ^b^	116.667 ± 9.465 ^d^	0.188 ± 0.004 ^b^	0.502 ± 0.047 ^c^
	60% ADSW	0.783 ± 0.102 ^bc^	49.048 ± 7.092 ^bc^	211.333 ± 15.543 ^a^	0.170 ± 0.037 ^b^	0.747 ± 0.049 ^b^
	80% ADSW	0.837 ± 0.075 ^bc^	52.619 ± 5.872 ^bc^	189.167 ± 7.638 ^b^	0.183 ± 0.030 ^b^	0.614 ± 0.172 ^bc^
	100% ADSW	0.852 ± 0.058 ^b^	53.929 ± 4.345 ^b^	205.833 ± 11.547 ^ab^	0.241 ± 0.001 ^a^	0.926 ± 0.009 ^a^
	BG11 medium	1.190 ± 0.062 ^a^	78.095 ± 4.185 ^a^	168.333 ± 11.547 ^c^	0.253 ± 0.004 ^a^	0.757 ± 0.034 ^b^
*C. vulgaris* FACHB-8	20% ADSW	0.778 ± 0.060 ^e^	51.786 ± 4.684 ^e^	141.667 ± 11.815 ^d^	0.189 ± 0.011 ^c^	0.614 ± 0.072 ^c^
	40% ADSW	0.933 ± 0.056 ^d^	62.857 ± 4.33 ^d^	106.667 ± 7.638 ^e^	0.185 ± 0.008 ^c^	0.424 ± 0.044 ^d^
	60% ADSW	1.140 ± 0.017 ^b^	77.619 ± 1.649 ^b^	295.000 ± 25.372 ^a^	0.198 ± 0.009 ^bc^	0.770 ± 0.075 ^b^
	80% ADSW	1.015 ± 0.045 ^c^	68.691 ± 2.865 ^cd^	239.833 ± 16.127 ^b^	0.218 ± 0.015 ^b^	0.852 ± 0.103 ^ab^
	100% ADSW	1.023 ± 0.038 ^c^	69.285 ± 3.093 ^c^	225.833 ± 2.887 ^b^	0.249 ± 0.007 ^a^	0.923 ± 0.039 ^a^
	BG11 medium	1.330 ± 0.035 ^a^	91.190 ± 2.909 ^a^	174.1667 ± 7.638 ^c^	0.264 ± 0.020 ^a^	0.755 ± 0.122 ^bc^
*Chlorella.* sp. FACHB-31	20% ADSW	0.697 ± 0.062 ^d^	46.190 ± 4.437 ^d^	95.000 ± 15.613 ^d^	0.142 ± 0.020 ^b^	0.364 ± 0.051 ^c^
	40% ADSW	1.080 ± 0.022 ^b^	73.572 ± 1.557 ^b^	161.667 ± 3.819 ^c^	0.178 ± 0.006 ^a^	0.292 ± 0.025 ^c^
	60% ADSW	1.118 ± 0.012 ^b^	76.310 ± 0.825 ^b^	273.333 ± 16.646 ^ab^	0.173 ± 0.031 ^a^	0.586 ± 0.049 ^b^
	80% ADSW	1.050 ± 0.046 ^b^	71.428 ± 3.273 ^b^	260.000 ± 8.660 ^b^	0.192 ± 0.009 ^a^	0.738 ± 0.052 ^a^
	100% ADSW	0.943 ± 0.040 ^c^	63.810 ± 2.509 ^c^	187.500 ± 9.014 ^c^	0.176 ± 0.007 ^a^	0.598 ± 0.035 ^b^
	BG11 medium	1.337 ± 0.040 ^a^	87.500 ± 6.814 ^a^	292.500 ± 27.839 ^a^	0.199 ± 0.007 ^a^	0.319 ± 0.019 ^c^

Data are expressed as mean ± SD (*n* = 3). Values with different letters represent significant difference (*p* < 0.05) between treatments.

**Table 3:** Removal of TN, TP, and NH_4_^+^-N by six microalgal strains in five dilutions of ADSW and BG11 medium within 14 days.

Algal Species	Culture Medium	TN			TP			NH_4_^+^-N		
		Initial (mg L^−1^)	Final (mg L^−1^)	Removal Efficiency (%)	Initial (mg L^−1^)	Final (mg L^−1^)	Removal Efficiency (%)	Initial (mg L^−1^)	Final (mg L^−1^)	Removal Efficiency (%)
*S. obliquus* FACHB-417	20% ADSW	52.43 ± 1.23	13.51 ± 0.70	74.24 ± 1.34 ^b^	14.21 ± 0.21	0.40 ± 0.05	97.19 ± 0.37 ^a^	37.46 ± 1.45	0.84 ± 0.03	97.75 ± 0.07 ^a^
	40% ADSW	105.71 ± 2.46	18.08 ± 1.25	82.89 ± 1.18 ^a^	26.87 ± 0.19	0.85 ± 0.12	96.85 ± 0.45 ^a^	73.60 ± 1.78	3.23 ± 0.15	95.61 ± 0.21 ^b^
	60% ADSW	153.80 ± 4.21	46.92 ± 1.12	69.48 ± 0.73 ^d^	39.60 ± 0.43	4.80 ± 0.14	87.87 ± 0.34 ^c^	111.34 ± 3.98	12.00 ± 0.84	89.22 ± 0.76 ^c^
	80% ADSW	205.80 ± 5.46	57.84 ± 0.71	71.91 ± 0.35 ^c^	53.46 ± 0.34	4.02 ± 0.03	92.47 ± 0.06 ^b^	147.87 ± 2.45	17.77 ± 1.89	87.98 ± 1.28 ^c^
	100% ADSW	258.50 ± 6.36	71.33 ± 0.91	72.41 ± 0.35 ^c^	65.75 ± 0.64	9.79 ± 0.18	85.12 ± 0.27 ^d^	183.50 ± 2.12	59.75 ± 1.17	67.44 ± 0.64 ^d^
*C. zofingiensis* GF1	20% ADSW	52.43 ± 1.23	13.15 ± 0.74	74.87 ± 1.40 ^a^	14.21 ± 0.21	5.39 ± 0.21	62.05 ± 1.44 ^b^	37.46 ± 1.45	13.22 ± 1.91	64.71 ± 5.10 ^a^
	40% ADSW	105.71 ± 2.46	29.28 ± 0.68	72.30 ± 0.64 ^b^	26.87 ± 0.19	15.57 ± 1.16	42.07 ± 4.31 ^d^	73.60 ± 1.78	26.23 ± 0.73	64.36 ± 1.00 ^a^
	60% ADSW	153.80 ± 4.21	59.06 ± 1.26	61.60 ± 0.82 ^d^	39.60 ± 0.43	16.82 ± 0.31	57.52 ± 0.78 ^c^	111.34 ± 3.98	55.42 ± 3.37	50.22 ± 3.03 ^b^
	80% ADSW	205.80 ± 5.46	69.61 ± 0.95	66.18 ± 0.46 ^c^	53.46 ± 0.34	18.76 ± 0.15	64.91 ± 0.28 ^b^	147.87 ± 2.45	71.97 ± 1.52	51.33 ± 1.03 ^b^
	100% ADSW	258.50 ± 6.36	84.03 ± 1.80	67.49 ± 0.70 ^c^	65.75 ± 0.64	19.76 ± 0.45	69.94 ± 0.68 ^a^	183.50 ± 2.12	91.17 ± 3.15	50.32 ± 1.72 ^b^
*C. protothecoides* GF2	20% ADSW	52.43 ± 1.23	10.59 ± 0.14	79.80 ± 0.26 ^b^	14.21 ± 0.21	2.38 ± 0.26	83.23 ± 1.85 ^d^	37.46 ± 1.45	2.00 ± 0.04	94.66 ± 0.12 ^b^
	40% ADSW	105.71 ± 2.46	17.43 ± 0.80	83.51 ± 0.75 ^a^	26.87 ± 0.19	0.65 ± 0.08	97.57 ± 0.28 ^a^	73.60 ± 1.78	0.74 ± 0.02	99.00 ± 0.03 ^a^
	60% ADSW	153.80 ± 4.21	41.31 ± 0.94	73.14 ± 0.61 ^d^	39.60 ± 0.43	4.42 ± 0.40	88.83 ± 1.00 ^b^	111.34 ± 3.98	24.83 ± 1.00	77.70 ± 0.90 ^c^
	80% ADSW	205.80 ± 5.46	53.41 ± 1.97	74.05 ± 0.95 ^d^	53.46 ± 0.34	6.29 ± 0.19	88.24 ± 0.36 ^bc^	147.87 ± 2.45	34.64 ± 0.86	76.57 ± 0.58 ^d^
	100% ADSW	258.50 ± 6.36	60.83 ± 1.09	76.47 ± 0.42 ^c^	65.75 ± 0.64	8.74 ± 0.16	86.71 ± 0.24 ^c^	183.50 ± 2.12	47.80 ± 0.98	73.95 ± 0.54 ^e^
*C. pyrenoidosa* GF3	20% ADSW	52.43 ± 1.23	25.90 ± 1.55	50.60 ± 2.96 ^d^	14.21 ± 0.21	1.09 ± 0.14	92.31 ± 1.02 ^b^	37.46 ± 1.45	1.45 ± 0.13	96.13 ± 0.35 ^b^
	40% ADSW	105.71 ± 2.46	32.51 ± 2.16	69.25 ± 2.04 ^c^	26.87 ± 0.19	1.36 ± 0.14	94.94 ± 0.54 ^a^	73.60 ± 1.78	1.81 ± 0.16	97.54 ± 0.22 ^a^
	60% ADSW	153.80 ± 4.21	38.00 ± 0.90	75.29 ± 0.58 ^b^	39.60 ± 0.43	2.69 ± 0.28	93.22 ± 0.71 ^b^	111.34 ± 3.98	17.88 ± 0.89	83.94 ± 0.80 ^c^
	80% ADSW	205.80 ± 5.46	48.14 ± 0.34	76.61 ± 0.16 ^b^	53.46 ± 0.34	5.04 ± 0.02	90.57 ± 0.04 ^c^	147.87 ± 2.45	21.87 ± 1.34	85.21 ± 0.91 ^c^
	100% ADSW	258.50 ± 6.36	52.62 ± 1.02	79.64 ± 0.39 ^a^	65.75 ± 0.64	7.91 ± 0.14	87.96 ± 0.21 ^d^	183.50 ± 2.12	36.07 ± 1.18	80.35 ± 0.65 ^d^
*C. vulgaris* FACHB-8	20% ADSW	52.43 ± 1.23	23.09 ± 0.08	55.95 ± 0.15 ^d^	14.21 ± 0.21	1.05 ± 0.14	92.63 ± 1.00 ^bc^	37.46 ± 1.45	1.67 ± 0.03	95.55 ± 0.09 ^b^
	40% ADSW	105.71 ± 2.46	27.95 ± 0.51	73.56 ± 0.48 ^c^	26.87 ± 0.19	1.24 ± 0.22	95.39 ± 0.81 ^a^	73.60 ± 1.78	1.62 ± 0.18	97.80 ± 0.25 ^a^
	60% ADSW	153.80 ± 4.21	32.85 ± 0.90	78.64 ± 0.59 ^b^	39.60 ± 0.43	1.87 ± 0.10	95.29 ± 0.26 ^a^	111.34 ± 3.98	8.38 ± 0.17	92.48 ± 0.15 ^c^
	80% ADSW	205.80 ± 5.46	41.90 ± 0.44	79.64 ± 0.21 ^b^	53.46 ± 0.34	3.57 ± 0.19	93.32 ± 0.36 ^b^	147.87 ± 2.45	12.73 ± 0.35	91.39 ± 0.24 ^d^
	100% ADSW	258.50 ± 6.36	40.85 ± 1.28	84.21 ± 0.49 ^a^	65.75 ± 0.64	5.13 ± 0.13	92.19 ± 0.20 ^c^	183.50 ± 2.12	16.22 ± 0.47	91.16 ± 0.25 ^d^
*Chlorella* sp. FACHB-31	20% ADSW	52.43 ± 1.23	18.48 ± 1.97	64.76 ± 1.76 ^d^	14.21 ± 0.21	0.77 ± 0.03	94.60 ± 0.21 ^b^	37.46 ± 1.45	2.26 ± 0.15	93.98 ± 0.40 ^b^
	40% ADSW	105.71 ± 2.46	25.94 ± 0.32	75.46 ± 0.32 ^c^	26.87 ± 0.19	0.98 ± 0.02	96.35 ± 0.07 ^a^	73.60 ± 1.78	1.54 ± 0.32	97.91 ± 0.43 ^a^
	60% ADSW	153.80 ± 4.21	35.33 ± 0.94	77.03 ± 0.61 ^bc^	39.60 ± 0.43	2.76 ± 0.15	93.03 ± 0.39 ^c^	111.34 ± 3.98	9.89 ± 0.42	91.12 ± 0.38 ^c^
	80% ADSW	205.80 ± 5.46	41.87 ± 2.36	79.66 ± 1.14 ^b^	53.46 ± 0.34	3.69 ± 0.31	93.10 ± 0.58 ^c^	147.87 ± 2.45	12.44 ± 0.47	91.59 ± 0.32 ^c^
	100% ADSW	258.50 ± 6.36	40.75 ± 0.59	84.24 ± 0.23 ^a^	65.75 ± 0.64	6.01 ± 0.14	90.89 ± 0.21 ^d^	183.50 ± 2.12	17.47 ± 0.52	90.48 ± 0.28 ^d^

Data are expressed as mean ± SD (*n* = 3). Values with different letters represent significant difference (*p* < 0.05) between treatments.

**Table 4:** Fatty acid composition (% total fatty acids) of FACHB-8 and FACHB-31 grown in undiluted ADSW after 15 days of cultivation.

Fatty Acid	*C. vulgaris* FACHB-8	*Chlorella* sp. FACHB-31
C14:0	8.52 ± 0.69	8.23 ± 1.09
C14:1	2.21 ± 0.03	2.37 ± 0.20
C16:0	27.46 ± 0.32	27.95 ± 0.37
C16:1	2.81 ± 0.13	2.40 ± 0.70
C18:0	17.59 ± 1.26	17.29 ± 0.82
C18:1 n-9	20.57 ± 0.07	20.25 ± 0.44
C18:2 n-6	16.61 ± 1.09	17.27 ± 0.11
C20:0	3.55 ± 0.86	3.58 ± 0.91
C20:5 n-3	0.67 ± 0.12	0.65 ± 0.10
SFA ^a^	56.74 ± 0.56	57.41 ± 0.50
UFA ^b^	43.26 ± 0.56	42.59 ± 0.50
MUFA ^c^	25.62 ± 0.04	24.65 ± 0.47
PUFA ^d^	17.65 ± 0.52	17.94 ± 0.03

^a^ SFA, saturated fatty acids. ^b^ MUFA, monounsaturated fatty acids. ^c^ UFA, unsaturated fatty acids. ^d^ PUFA, polyunsaturated fatty acids.

## Data Availability

The data of this study are available from the correspondence author upon reasonable request.
